# Reframing Child Protection in Emergency Medicine

**DOI:** 10.5811/westjem.18481

**Published:** 2024-10-01

**Authors:** Joseph P. Shapiro, Genevieve Preer, Caroline J. Kistin

**Affiliations:** * Children’s National Hospital, Department of Emergency Medicine and Trauma, Washington DC; † Boston University Chobanian & Avedisian School of Medicine, Boston, Massachusetts; ‡ Boston Medical Center, Boston, Massachusetts; § Brown University, School of Public Health, Hassenfeld Child Health and Innovation Institute, Department of Health Services, Policy, and Practice, Providence, Rhode Island

## Abstract

Child maltreatment remains a concerning source of morbidity and mortality in the United States, where more than 600,000 children are victims of abuse each year, with well-described, long-term consequences for physical and mental health.

However, the US child welfare system is characterized by systemic racism and inequity. Black and Native American children are more likely to be evaluated and reported for suspected abuse despite evidence that race does not independently change their risk of being abused. Once reported to child protective services (CPS), these children are more likely to be removed from their homes and less likely to be reunited with their families than White children.

Much of the inequity in this system starts at the front door, where a growing body of research demonstrates that bias regularly infiltrates decision-making in the initial clinical evaluation and management of suspected abuse. Minority children presenting to emergency departments (ED) are more likely to receive diagnostic testing and are more likely to be referred to CPS.

In this editorial, we argue for the application of an equity lens to child protection in the ED. We discuss how emergency physicians can balance efforts to protect children from abuse with the imperative to protect children and families from the harms of an inequitable child welfare system. Our discussion concludes with concrete recommendations for emergency clinicians to participate in active bias mitigation and thoughtfully navigate their responsibilities as mandated reporters.

Child maltreatment remains a concerning cause of morbidity and mortality in the United States, where greater than 600,000 children are victims of substantiated abuse each year with long-term consequences for physical and mental health.^1^ The US child welfare system, however, is characterized by marked inequity.^2^ ^,^ ^3^ Black and Native American children are more likely to be evaluated and reported for suspected abuse despite evidence that race does not independently change their risk of being abused.^4^ ^,^ ^5^

In years past, typical clinical decision-making might have dictated that even a small suspicion of child abuse meant an automatic report to child protective services (CPS). However, with the growing recognition of the racial inequity within the child welfare system and its adverse effects on communities of color, emergency physicians now must reconsider their approach.^6^ ^–^ ^8^ As frontline care clinicians for injured children, emergency physicians should seek to strike a balance between safeguarding children from abuse and shielding families from unnecessary exposure to the child welfare system.

In a systematic review, Cenat et al compiled 36 studies of proportionality in child welfare systems and found that 27 of these studies show Black youth are over-represented in these systems.^2^ This disproportionality is present at each phase of the child protection process beginning with reporting. Nationally, Black children represent 15% of the general population but account for 25% of CPS investigations.^9^ Separate state-level analyses of public records in Washington, Colorado, Missouri, Wisconsin, Texas, and North Carolina show that Black children are at least twice as likely as White children to be reported to CPS.^10^ ^–^ ^15^

Once an investigation is initiated, Black and Native American children are more likely to be removed from their homes and less likely to be reunited with their families than White children.^16^ In a record review of 4,000 children referred to CPS in California, Lu et al found that in comparison to White children, Black children were 23.4% more likely to be placed in out-of-home care and 11.7% less likely to be reunified with their families if removed from the home.^17^ A similar review by the Center for the Study of Social Policy found that Black victims of maltreatment were 36% more likely than White victims to be removed from their homes.^18^

The burden of child welfare involvement on Black families and communities is substantial. Research by Kim et al indicates that over 50% of Black children will undergo a CPS investigation within their family before turning 18.^19^ Sociologist Dorothy Roberts and a coalition of academics and community advocates lead an intellectual movement that characterizes the child welfare system as a form of “family policing.” Through a robust body of qualitative and narrative research they highlight how the system disrupts parental authority, hinders children’s relationship development, fosters distrust among neighbors, and harms maternal mental health.^7^ ^,^ ^20^ ^–^ ^21^ There is little evidence that contact with the child welfare system can improve outcomes for children or families.^22^

The role of emergency physicians as mandated reporters and providers of frontline care to injured children makes them de facto gatekeepers to this unequal system. Most CPS referrals in hospitals are for patients whose care starts in the emergency department (ED).^23^ Both general emergency physicians and pediatric emergency physicians care for children who may have been the victims of physical or sexual abuse, or who may be experiencing one of multiple types of neglect (physical, supervisory, medical, educational, and emotional). A growing body of research demonstrates that bias regularly infiltrates decision-making in the initial clinical evaluation and management of suspected abuse. In a secondary analysis of CPS reports conducted at an academic tertiary hospital, Cort et al found Black and Hispanic pediatric patients were four times more likely than White pediatric patients to be reported to CPS by medical personnel.^24^

Lane et al demonstrated in a retrospective cohort study that Black and Latinx children seen for fractures were eight times more likely than White children to receive a skeletal survey and four times more likely to be reported to CPS, regardless of insurance status (poverty) or likelihood of an abusive injury.^5^ In a study of pediatric patients admitted for traumatic brain injury at 39 hospitals, Wood et al observed that fewer skeletal surveys are performed among White infants (67.8%) than Black infants (84%).^25^ In a cross-sectional analysis from 18 sites in the Pediatric Brain Injury Research Network, Hymel et al found that among acutely head-injured patients hospitalized for intensive care, minority patients were twice as likely to be evaluated and reported for abuse than White patients. This disparity was seen most in patients who had a low estimated risk of abusive head trauma at the outset of the encounter.^26^ Most recently, Rebbe et al showed that among hospitalized patients, Asian and Native American children were more likely than White children to have their encounter coded with a maltreatment-related diagnostic code and to be reported to CPS.^27^ It is in part because bias factors into child maltreatment reporting that emergency physicians have such a meaningful role to play.

It is not our intention to encourage physicians to shirk mandated reporting requirements. Physicians with a “reasonable suspicion” of child maltreatment should report their concerns to the appropriate authorities. This remains the legal standard in most jurisdictions across the United States. That said, it must be acknowledged that considerate, well-trained people disagree on what constitutes “reasonable suspicion.” The term is plagued by indeterminacy in theory and practice.^28^ Survey studies consistently show wide variation among physicians attempting to define reasonable suspicion and reach consensus on the threshold for reporting.^29^ In one study, clinicians chose to report only 73% of children they considered “likely” or “very likely” to have been abused,^30^ while in a separate study nearly half of respondents said they would report cases where abuse was fifth or lower on their list of potential differential diagnoses.^31^ In many cases, it cannot be assumed that any two mandated reporters agree on the need for a CPS report.

In the setting of this indeterminacy, the burden falls to the individual clinician, and it is arguably an ethical one more than a legal one. Unnecessary reports to CPS stand in direct conflict with the principles of non-maleficence and justice. Reasonable people may disagree on the threshold for reporting potential abuse, but all clinicians should consider the full scope of harms and benefits any time they contemplate a filing.

Optimistically, this challenge is also an opportunity. There is a growing enthusiasm for remedying the child welfare system among pediatric healthcare clinicians in many settings.^4^ ^,^ ^6^ Emergency and pediatric emergency physicians are perfectly positioned to join this movement and make positive changes in an unjust system. In the recommendations that follow, we draw on the work of experts in child protection and implicit bias to suggest interventions for emergency physicians at the intrapersonal and interpersonal levels.

1. Stay abreast of current evidence and recommendations. The evidence base for child abuse pediatrics is rapidly evolving. It is increasingly understood which mechanisms are feasible explanations for certain injuries, making it possible to accurately identify the findings most indicative of abuse. A good understanding of this evidence base allows clinicians to avoid unnecessary workups and referrals. The American Academy of Pediatrics provides clinical practice guidelines that summarize current recommendations for the evaluation of physical abuse.^32^ ^,^ ^33^ Going a step further, physicians can use their knowledge of evidence-based practice to implement standardization within their departments. Limited studies show that clinical practice guidelines can improve disproportionality in child maltreatment evaluation.^34^ This should be done carefully. Standard screening guidelines may improve proportionality, but standardized referral guidelines risk overexposing children and families of all races to the child welfare system.
2. Use a multidisciplinary approach to child protection. A thorough protective assessment by a skilled social worker is an invaluable tool for determining whether a CPS filing is necessary. In addition to the injury history typically obtained by the medical team, social workers often have the opportunity to learn more about family dynamics, housing status, caretaking responsibilities, protective factors, or prior CPS involvement. All this information can be used to make a holistic assessment of protective concerns and inform dialogue to mitigate bias.
3. Consult child abuse pediatricians whenever possible. Child abuse pediatricians are subspecialists with extensive training and expertise in all aspects of child protection. They can recommend diagnostic workups, comment on whether injuries are consistent with stated mechanisms, and help physicians navigate the reporting process. There is evidence that consultation with a child abuse pediatrician can reduce unnecessary referrals to CPS.^35^
4. Understand your local CPS and make referrals in accordance with their capabilities. A good emergency room physician does not order a test or treatment without knowledge of its limitations. The same should apply to a report to CPS. What types of concerns do investigators have the expertise to investigate? What supports can they offer to families? Can they help with childcare, housing, transportation, or employment? It is a common misconception among clinicians that a report to CPS benefits families by helping them to access additional resources.^36^ ^,^ ^37^ In practice, it is uncommon for CPS to provide social supports that could not have been made available by a hospital social worker or primary pediatrician. It has been questioned whether CPS has an impact at all on modifiable risk factors for abuse. In a multicenter cohort study of 595 children between 4–8 years old judged to be at risk of abuse, Campbell et al found no association between CPS investigation and subsequent social support, family function, poverty, maternal education, or child behavioral problems.^38^
5. Be careful about language and information sharing. When communicating about a case with colleagues or CPS, avoid discussing unnecessary demographic details (race, employment status), or sharing subjective judgments (“lovely family”). The more communication is limited to essential information and neutral language, the more we can minimize the effect of bias.

Emergency clinicians can play an essential role in decreasing systemic inequity in child welfare by mitigating implicit and explicit bias and taking a thoughtful approach to mandated reporting.
